# Chromic Acid

**Published:** 1869-10

**Authors:** 


CHROMIC ACID.—Dr. E. Magitot (*Bull Gen. de la Ther.*) especially recommends chromic acid in the affection, known as "alveolo-dental osteo-periostitis." He also advocates it in all forms of stomatitis, aphthæ, and other ulcerations of the buccal mucous membrane.

